# University students’ free time management and quality of life: the mediating role of leisure satisfaction

**DOI:** 10.1186/s40359-024-01745-2

**Published:** 2024-04-27

**Authors:** Esranur Terzi, Utku Isik, Berat Can Inan, Can Akyildiz, Umit Dogan Ustun

**Affiliations:** 1 Graduate School of Education, Recep Tayyip Erdogan University, Rize, Türkiye; 2 Faculty of Sport Sciences, Ege University, Izmir, Türkiye; 3 Faculty of Sport Sciences, Recep Tayyip Erdogan University, Rize, Türkiye

**Keywords:** Activity, Exercise, Recreation, Free time, Life satisfaction

## Abstract

The impact of free time management and leisure satisfaction on quality of life is distinct, however, the role of satisfaction in enhancing quality of life through free time management remains uncertain. Hence, the objective of this research is to explore how leisure satisfaction acts as a mediator between free time management and the levels of quality of life among university students. Additionally, this study aims to analyse these concepts in relation to gender, age and the number of days of activity participation. Within this particular framework, a total of 213 university students willingly participated in the survey, which included the administration of the “Free Time Management Scale,” “Leisure Satisfaction Scale,” and “Quality of Life Scale.” The analyses employed the Independent T-Test, Pearson Correlation, and Linear Regression methods. The mediating effect was analysed using Structural Equation Modelling. The study found significant relationships between gender, free time management, and life quality. There was a significant relationship between free time management, leisure satisfaction, and quality of life (*p* < 0.05). Leisure satisfaction partially mediated the quality of life-free time management relationship. As age and physical activity grow, males have a higher standard of living, and time allocation and quality of life improve. Furthermore, it was found that students who effectively managed their time experienced an enhanced quality of life, as evidenced by their increased satisfaction with leisure activities. Notably, the level of satisfaction with well-managed time was identified as a crucial factor in this association.

## Introduction

Engaging in leisure activities, which are now recognized as essential for both mental and physical well-being, is highly crucial for individuals to maintain a healthy lifestyle. The repetitive nature of one’s lifestyle and mundane daily routines can lead to many mental and physical issues. Consequently, scholars [1–3] have directed their attention on examining the free time activities, life satisfaction, and physical activities undertaken during leisure by individuals from diverse viewpoints. Leisure holds significance for numerous individuals, serving as a source of enjoyment and a means of evading external pressures imposed upon them [4]. In order to gain a deeper comprehension of how free time affects individuals, researchers frequently analyze the level of contentment individuals experience during their leisure activities. This analysis encompasses various elements such overall life satisfaction, quality of life, effective management of free time, limitations on leisure activities, satisfaction with one’s community, and the ability to effectively manage stress [4].

Free time management examines how individuals organize their free time and the effects of this process. This concept encompasses the effective and efficient use of time, achieving goals, and maintaining balance. Effective free time management can help individuals reduce stress, maintain balance, and enhance their quality of life [5]. Leisure satisfaction investigates how individuals assess their leisure and how these experiences affect their quality of life. It represents the level of enjoyment individuals derive from engaging in activities they enjoy. Sufficient leisure satisfaction can alleviate stress, enhance psychological well-being, and increase overall life satisfaction [1]. Quality of life refers to individuals’ overall satisfaction and well-being in life. This concept is shaped by a combination of physical, psychological, social, and environmental factors. A high quality of life enables individuals to feel generally happier, healthier, and more fulfilled [5].

### Mediator role of leisure satisfaction in the effect of free time management on quality of life

*Free time management* examines an individual’s allocation of free time and assesses if they fulfil their spiritual and physical requirements during this period. Free time activities are crucial for the working class and students, who have less free time compared to other segments of society, since they help alleviate tension and fatigue resulting from their job life [1]. The rationale for prioritizing this domain lies in the fact that effectively utilizing one’s free time can enhance personal happiness and foster greater success in social interactions. Furthermore, numerous scholars have highlighted that quality of life is a multifaceted notion that necessitates both objective and subjective measurements [6]. Quantifiable measures of quality of life encompass factors such as the state of one’s living surroundings, physical well-being, degree of income, and socioeconomic standing [6]. On the other hand, subjective of quality of life encompass factors such as overall living conditions, life satisfaction, happiness, and personal contentment [1]. Effective management of free time enhances the quality of life by positively impacting participation, satisfaction, attitudes, health, and environment [5]. Previous studies have discovered a direct correlation between effectively managed free time and engagement in physical activities, as well as an improved quality of life in terms of health [7]. Effectively managing free time is a fundamental factor that enhances one’s quality of life. Thus, the quality of life is inherently connected to the conceptual aspects of effectively managing one’s free time and experiencing enjoyment in leisure activities. Free time management enables individuals to utilize their free time in purposeful activities, foster resilient communities, pursue favourable psychological well-being, acquire novel proficiencies, and eventually enhance their quality of life [6].

While there is evidence suggesting a positive connection between leisure satisfaction and free time management [5], researchers have not yet agreed on the precise nature of the relationship between different aspects of free time management and quality of life. When examining free time, researchers distinguish between two types of leisure variables: person-centered and place-centered [8]. Person-centered leisure variables include leisure participation, satisfaction, and attitude, while place-centered leisure variables encompass leisure resources and environment. Lloyd and Auld [8] argue that both leisure variables, person-centered and place-centered, need to be measured when assessing leisure activities, while findings by Leung and Lee [9] indicate that the interaction between person-centered and place-centered leisure activities creates and sustains life quality. Passmore and French [10] have identified three types of leisure activities in which adolescents participate: achievement-oriented leisure activities, social leisure activities, and time-passing leisure activities. Lloyd and Auld [8] have categorized leisure activities into six groups based on their frequencies: mass media, social activities, outdoor activities, sports activities, cultural activities, and hobbies. Scott and Willits [11] reported four types of leisure activities classified as socializing, creative or artistic, intellectual, and physical activities. Although there is no consensus in the literature regarding the classification of leisure activities, researchers generally agree on the contribution of leisure to life quality and suggest that the relationship between leisure and life quality is complex [12, 13]. Various explanations underlie the relationship between leisure and life quality in the literature. According to activity theory, higher participation frequencies and more meaningful activities are associated with higher levels of life quality [13]. Previous studies have shown a positive relationship between participation in physical leisure activities and life quality [9], as well as health-related quality of life [7]. Additionally, Robinson and Martin [14] have shown that most happy individuals are more active in social activities. An alternative theoretical framework is the needs theory, which posits that meeting needs has beneficial effects on life quality [13]. The aforementioned studies specifically reported that the higher individuals perceive their needs for satisfaction and participation in recreation, the higher their quality of life. Hence, an additional objective of this essay is to elucidate the impact of effective free time management on one’s quality of life.

To explain this effect, we can examine theories such as the “Boundary Theory” and the “Psychological Separation Theory.” The Boundary Theory examines the balance between work and free time, focusing on how individuals organize their lives. According to this theory, a harmonious balance between work and leisure can enhance individuals’ quality of life [15]. Leisure activities can alleviate stress stemming from work life, thereby increasing overall life satisfaction. For instance, engaging in free time activities allows individuals to distance themselves from work-related stressors, facilitating mental and emotional relaxation and rejuvenation [15]. On the other hand, the Psychological Separation Theory emphasizes the importance of delineating clear boundaries between work and free time. According to this theory, establishing distinct boundaries between work and leisure prevents individuals from carrying work-related stress into their free time, thus enhancing quality of life. Particularly for university students, maintaining a clear separation between study periods and free time activities can improve academic performance and enhance overall life satisfaction [16, 17]. By integrating these theories, we can better understand the relationship between free time management and quality of life. Leisure activities not only facilitate coping with work-related stress but also provide opportunities for psychological relaxation, ultimately enhancing individuals’ quality of life [18].

Given the aforementioned linkages, we formulated a hypothesis for a model that could elucidate the connections between quality of life and the management of free time:

- *Hypothesis 1: The free time management has a positive impact on the quality of life of university students.*

*Leisure satisfaction* is the favourable thoughts or sensations that individuals have when they engage in leisure activities that align with their preferences, successes, and expectations. Essentially, it refers to the level of contentment that an individual experience from their leisure activities [2]. This felt fulfilment arises from fulfilling the demands that the individual perceives as deficient or believes are not being fulfilled [3].

Leisure satisfaction is frequently regarded as a higher priority compared to other factors such as economic and social status, security, and religion [19]. According to Agate et al. [20], leisure satisfaction was found to be the most accurate predictor among criteria such as family income, age, married status, and leisure involvement in determining family life satisfaction. Prior research has also emphasized the favourable association between satisfaction with leisure activities and the quality of life [21]. For instance, Chun et al. [22] discovered that a significant degree of contentment with leisure activities can mitigate stress, but a smaller degree may be linked to an unhealthy way of life. While there is a positive correlation between leisure satisfaction and quality of life, the exact nature of the relationship between different components (happiness or peacefulness) of leisure satisfaction and quality of life is still a topic of debate among academics [23]. Hence, the primary objective of this article is to elucidate the impact of leisure satisfaction on the quality of life.

We can examine this effect through theories such as the “Leisure Satisfaction Theory” and the “Social Resources Theory”. The Leisure Satisfaction Theory explores how individuals utilize their leisure and how these experiences affect their quality of life. Leisure satisfaction refers to the level of enjoyment an individual derives from engaging in activities they enjoy. Research indicates that adequate leisure satisfaction enhances quality of life. A satisfying leisure experience can reduce stress, enhance psychological well-being, and elevate overall life satisfaction [24]. This theory can be utilized to explain the quality of life among university students by focusing on how students utilize their leisure and how these experiences contribute to their overall life satisfaction. On the other hand, the Social Resources Theory examines how individuals can enhance their quality of life through social relationships and resources. For university students, social support, friendships, and family bonds are crucial. Social resources play a critical role in coping with stress and improving quality of life [25]. This theory can also be applied to explain the quality of life among university students. Social support networks, leisure activities, and friendships can positively influence students’ quality of life. By integrating these theories, we can better understand the impact of leisure satisfaction on quality of life among university students. Leisure activities not only enhance individuals’ personal satisfaction but also promote social interactions, thereby increasing access to social resources. This interaction plays a significant role in improving overall quality of life [26]. This integration allows us to better comprehend the multifaceted effects of leisure activities on quality of life and provides a more comprehensive interpretation of research findings.

Given the aforementioned linkages, we formulated a hypothesis for a model that could elucidate the connections between quality of life and satisfaction derived from leisure activities:

- *Hypothesis 2: The leisure satisfaction has a positive impact on the quality of life of university students.*

*Mediator* The purpose of these studies [1–3] is to investigate the subjective enjoyment that individuals derive from their lives, specifically focusing on how to optimize their overall well-being. Quality of life refers to the fulfilment of one’s aspirations, taking advantage of possibilities for personal growth, engaging in diverse activities, possessing adequate resources in terms of quality, and perceiving these resources as satisfactory [27].

It may be inferred that free time management in leisure activities might lead to increased enjoyment and improved quality of life for individuals [28]. Chick et al. [4] verified that inadequate time management during free periods had a detrimental impact on the level of satisfaction derived from leisure activities. Within this framework, individuals who effectively allocate their time and structure their lives in alignment with their personal requirements, resulting in the experience of happy emotions rather than negative emotions, may exhibit elevated levels of subjective well-being and life satisfaction [29]. Therefore, individuals who have a high quality of life are more likely to meet their needs effortlessly, have control over their surroundings, exercise their autonomy in decision-making, have opportunities for personal growth, and lead a purposeful existence [30]. In general, the researchers discovered a positive correlation between leisure satisfaction and quality of life [31]. In their study, Spiers & Walker [23] found a strong correlation between leisure satisfaction and eight aspects of quality of life. These aspects include happiness, well-being, living standards, health, achievement, personal relationships, community involvement, and spirituality. Research has additionally demonstrated that effective control of free time plays a crucial role in managing the quality of life [28]. Generally, there is a favourable correlation between free time management and satisfaction with leisure activities, both of which contribute to quality of life. While prior research has examined the correlation between the management of free time and satisfaction with leisure activities, as well as the quality of life, there has been a lack of attention given to the impact of leisure satisfaction on the connection between free time management and quality of life.

Links have been found between leisure and life satisfaction, subjective well-being, and quality of life [32, 33]. Leisure or its absence is associated with “lifestyle diseases,” particularly obesity, stress, and depression [34]. Other studies indicate that leisure reduces stress [35], enhances mood [36], and contributes to overall health and well-being [37]. Leisure participation and leisure satisfaction are associated with life satisfaction [38]. For example, Spiers and Walker [23] argue that “leisure satisfaction is likely the best predictor of happiness and quality of life.” In summary, leisure seems to contribute multifacetedly to perceived quality of life and individual life satisfaction [39]. On the other hand, leisure constraints, as defined by Jackson [40] as things or conditions that impede people from participating in leisure activities, spending more time doing so, benefiting from leisure services, or achieving a desired level of satisfaction are generally acknowledged to have negative effects on aspects of life quality including leisure participation, leisure satisfaction, emotional well-being, and health [23]. Ngai [41] found leisure satisfaction to be significantly associated with measures of quality of life in Macao, China. Hawkins et al. [42] found that although the impact of leisure satisfaction was substantially greater than other variables in both cases, leisure constraints in samples from Australia and the US were associated with life satisfaction, leisure satisfaction, and leisure activity participation. Mannell & Dupuis [43] found evidence of a positive relationship between physical leisure activity and life satisfaction.

The “Leisure Satisfaction Theory” provides a suitable framework to elucidate the relationship between university students’ quality of life and their free time management. This theory examines how individuals assess their free time and how these experiences affect their overall quality of life. Leisure satisfaction refers to the degree to which individuals enjoy engaging in activities they prefer. Research indicates that sufficient leisure satisfaction enhances quality of life [44]. A high level of leisure satisfaction can alleviate stress, enhance psychological well-being, and elevate overall life satisfaction [45]. This theory offers a pertinent framework to explain university students’ quality of life because it focuses on how students evaluate their free time and how these contribute to their overall life satisfaction. Furthermore, the “Social Psychology of Time” theory can also be instrumental in explaining this relationship. This theory explores the social and psychological dimensions of time and emphasizes the impact of time use on individuals’ quality of life. The time management of young adults, such as university students, can affect their quality of life based on their social interactions, personal development, and relaxation needs [46]. By integrating these theories, we can better understand the effects of university students’ free time management on their quality of life. Research conducted within this integrated theoretical framework can provide detailed insights into the effects of university students’ free time management on their quality of life. This, in turn, can enhance our understanding of this relationship and facilitate the development of effective interventions aimed at improving university students’ quality of life. The strong direct effects of leisure satisfaction on life satisfaction and indirect effects on self-rated health suggest that other leisure-related variables such as leisure motivations, attitudes toward leisure, and social support networks related to leisure activity could be significantly associated with life satisfaction and self-rated health. To discover the key elements influencing quality of life, it is crucial to analyze the role of leisure pleasure in the relationship between free time management and quality of life, given that these two factors have distinct impacts on quality of life.

Given the aforementioned associations, we formulated a hypothesis for a model that could elucidate the role of leisure pleasure in mediating the connection between quality of life and free time management:

- *Hypothesis 3: The relationship between the management of university students’ free time and their quality of life is mediated by their satisfaction with leisure activities.*

### The impact of age, gender, and participation in activities on the management of free time, satisfaction with leisure, and quality of life

Several factors can impact the time management, satisfaction with leisure activities, and quality of life of individuals. These variations encompass disparities in age, gender, profession, level of physical well-being, societal standing, and life responsibilities [47]. Upon reviewing the literature, it is appropriate to utilize social theories and psychological models to assess the effects of age, gender, and participation in activities on free time management, leisure satisfaction, and quality of life [48–51]. For instance, the impact of age and gender on free time management can be elucidated through social structure theories, while the influence of activity participation on leisure satisfaction and quality of life can be examined using psychological models. Social structure theory focuses on individuals’ roles and relationships within the social structure. Demographic factors such as age and gender influence social structure and consequently shape free time management [48, 51]. For example, individuals belonging to different age groups may have varied social roles and responsibilities, which affect how they allocate their leisure. Gender, on the other hand, is associated with societal gender roles and expectations, which can influence leisure activities and time management [48]. In this context, research can assess the effects of age and gender on free time management to test social structure theory. Psychological models, on the other hand, focus on individuals’ internal processes and motivations to explain behavior [49, 50]. They can be utilized to evaluate the effects of activity participation on leisure satisfaction and quality of life. For instance, individuals’ motivations and emotional experiences related to their participation in activities can affect leisure satisfaction. Engaging in specific activities can fulfill individuals’ emotional and psychological needs, thereby enhancing their quality of life [49]. In this framework, research can conduct tests to understand the effects of activity participation on leisure satisfaction and quality of life using psychological models [50]. The utilization of these theoretical models can assist in comprehensively understanding the effects of age, gender, and activity participation on free time management, leisure satisfaction, and quality of life. In the literature, Bernard and Phillipson [52] examined the relationship between age and leisure satisfaction, finding a decrease in satisfaction with increasing age. According to Dixon [53] research, women experience a lack of enjoyment when it comes to leisure activities. Ateca-Amestoy et al. [54] discovered that social factors have a more significant impact on leisure satisfaction compared to economic factors. Francken & Raaij [55] discovered a positive correlation between age and leisure satisfaction, indicating that older individuals experienced greater satisfaction in their leisure activities compared to younger individuals. Conversely, Su et al. [56] noticed a negative relationship between age and leisure satisfaction, suggesting that older people were less content with their leisure activities in comparison to younger people.

Research on gender differences in free time management [57] suggests that women encounter growing limitations in terms of structure, relationships, and personal factors. Interpersonal limitations persist in sports and leisure domains due to their predominantly male-dominated nature [58]. Furthermore, women have been reported to be significantly burdened by interpersonal limitations. For instance, Wilson & Little [58] discovered that women had greater limitations in engaging in leisure sports activities compared to men. According to a separate study conducted by Demir and Alpullu [59], it was found that the way free time is managed differs among various age groups. Moreover, prior studies have consistently revealed gender disparities indicating that females typically have a worse standard of living in comparison to males. Studies by Lassander et al. [60] indicate that men have a superior quality of life, particularly in terms of their physical and psychological well-being. In addition, research conducted by Lassander et al. [60] reveals that the impact of quality of life on individuals is universally utilizing and diminishes as they grow older.

Given the aforementioned associations, we formulated a hypothesis for a model that could elucidate the impact of gender, age, and engagement in activities on quality of life, time management, and pleasure with leisure:

- *Hypothesis 4: The quality of life, management of free time, and satisfaction with leisure activities among university students vary according on their gender, age, and level of engagement in activities*.

## The present study

*The Importance of Quality of Life:* The quality of life of university students is a significant indicator during young adulthood, a period characterized by intensive personal and academic development. Therefore, understanding the quality of life of university students is a critical step in assessing their overall well-being and achievements. *The Importance of Free Time Management:* University students are required to allocate time not only to academic studies but also to social activities and personal interests. Hence, skills in leisure time management are important for university students. Effective leisure time management can help cope with stress, maintain balance, and enhance overall quality of life. *The Role of Leisure Satisfaction:* Leisure satisfaction focuses on how individuals assess their leisure time and how these experiences affect their quality of life. In this context, investigating university students’ leisure satisfaction can help us understand its effects on their overall life satisfaction.

Previous research [57–60] has often been limited in scope, focusing on specific aspects, and has not fully addressed the relationship between leisure time management and quality of life among university students as comprehensively as our study aims to do. Some studies have only examined certain variables to explain the relationship between leisure time management and quality of life, which may not fully reflect the complexity and multifaceted nature of the relationship. The mediating role of leisure satisfaction in the relationship between leisure time management and quality of life has also been understudied. Therefore, it is important to conduct more comprehensive research to fully understand the relationship between leisure time management and quality of life among university students. This research can contribute to the development of strategies to improve the quality of life of university students.

Examining free time management, leisure satisfaction, and quality of life in a sample of university students provides insights into the mental well-being and perspectives of our future young folks. Given that the actions undertaken during university education influence individuals’ future conduct and the societal context, it is crucial to ascertain the degree to which the university students in our study may regulate their allocation of free time to engage in various activities.

Hence, the objectives of this article are two-fold: (1) to examine how leisure satisfaction influences the connection between university students’ management of free time and their quality of life, and (2) to assess whether demographic variables serve as significant predictors of free time management, leisure satisfaction, and quality of life.

Our study holds significance in giving a novel and current source to the literature by investigating the free time management, leisure satisfaction, and quality of life among university students of all genders and age groups. Furthermore, it is crucial to completely assess free time management, leisure satisfaction, and quality of life within the chosen sample, with the aim of providing guidance to educators in mitigating any mental and physical health issues.

## Materials and methods

### Participants

**Fig. 1:**
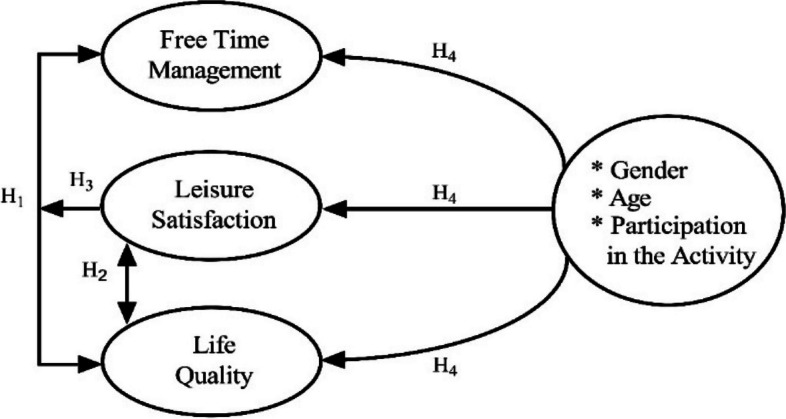
Model of the study

The study was designed as a quantitative cross-sectional study, utilizing a survey method for data collecting. In the study, by revealing the relationship between university students’ free time management, leisure satisfaction and quality of life, it was determined whether demographic characteristics such as gender, age and the number of days of activity participation affect free time management and leisure satisfaction. The framework of this conceptual model is shown in Fig. 1.

This study is a cross-sectional research conducted between November 2022 and December 2022, in which university students actively enrolled during this period were selected through simple random sampling. Selected students met the inclusion criteria and were administered an electronic survey followed by face-to-face interviews. Prior to participation, students were informed about the purpose, procedures, and requirements of the survey, and provided informed consent by signing a consent form after fully understanding the study. Data were collected within 2 weeks only from those who volunteered to participate. This survey encompasses both male and female university students. The sample size was determined using G*Power software. Using a priori analysis, we determined that a sample size of 174 individuals was necessary. This calculation was made with a power of 0.95 and an effect size of 0.55. The sample calculation followed the procedures recommended by Serinolli and Novaretti [61]. A total of 213 individuals, selected through random sampling based on volunteers, participated in the study. Thus, in the study, the principle of giving weight to large samples in structural equation modeling studies, and basing on a minimum of 15 cases per indicator, has been taken into account [62]. The participants had an average age of 23.61 ± 5.84. Participants who did not meet any of the criteria specified below were excluded from the study:

- Age range: 18–35 years
- Pursuing higher education at a university
- Voluntarily participation

## Data collection

The research data were gathered utilizing the “Personal Information Form,” “Free Time Management Scale,” “Leisure Satisfaction Scale,” and “Life Quality Scale”. The survey consisted of two sections; the first section pertained to explaining the scope of the research and collecting demographic information. The second section comprised 51 questions related to the main variables of the study. The researcher requested participation in the survey from 213 participants. Since there were no missing data, responses from these 213 surveys were utilized for analysis.

## Data collection tools

### Personal information form

The researchers developed this form in order to gather information on several independent variables, including gender, age, height, weight, and the frequency of participation in the activity. All variables included in this form were selected based on previous research [57].

### Free time management scale

The free time management scale to be used in the study was developed by Wang et al. [63]. Wang et al. [63] utilized confirmatory factor analysis (CFA) to assess the measurement model. The results of the CFA indicate that all standardized loadings exceeded 0.57. With regard to the goodness of fit of the model, the χ2 statistic was 183.41 with 83 degrees of freedom (*P* < 0.01). the goodness of fit index (GFI) was 0.94, the root mean square error of approximation (RMSEA) was 0.06, the adjusted goodness of fit index (AGFI) was 0.92, the normalized fit index (NFI) was 0.95, the comparative fit index (CFI) was 0.97, and the standardized root mean square residual (SRMR) = 0.05.

The scale was adapted into Turkish by Akgul & Karakucuk [64]. The scale consists of 15 items. Items on the scale are rated on a 5-point Likert scale as follows: 1: Strongly Disagree, 2: Disagree, 3: Neutral, 4: Agree, 5: Strongly Agree. Thus, higher scores indicated more positive free time management. Three statements in the scale questions are reverse coded. In the adaptation study of the scale, the internal consistency coefficient was calculated as .83 in the total sample. In our sample, the reliability coefficient of the scale was calculated as .86 in total.

### Leisure satisfaction scale (LSS)

The LSS, developed by Beard & Ragheb [65], was adapted into Turkish by Gokce & Orhan [66]. In the research conducted by Beard & Ragheb [64], the results of CFA revealed the following statistics: model χ2 = 12.54 (df = 6, *p* = 0.051); RMSEA = 0.025; CFI = 1.00; AGFI = 0.98; and SRMR = 0.018.

The LSS consists of 24 items. Items on the scale are scored as “Almost Never True (1)”, “Rarely True (2)”, “Sometimes True (3)”, “Often True (4)”, and “Almost Always True (5)”. Thus, higher scores indicated more positive leisure satisfaction. In the adaptation study of the scale, the internal consistency coefficient was calculated as .90 in the total sample. In our sample, the reliability coefficient of the scale was calculated as .93 in total.

### Quality of life scale (SF-12)

The quality-of-life scale is a shortened version of the SF-36 scale, which was created by Ware et al. [67]. The 12-item version of the scale, which was translated into Turkish by Soylu & Kutuk [68], was employed in our study. Items 1, 8, 9, and 10 of the scale are coded in reverse. Items related to physical and emotional roles are answered as yes or no, while other items have Likert-type options ranging from 3 to 6. A higher score from the scale indicates better health. In the adaptation study of the scale, the internal consistency coefficient was calculated as 0.73 in the total sample. In our sample, the reliability coefficient of the scale was calculated as 0.71 in total.

## Data analysis

The statistical analyses were performed using IBM SPSS Statistics 27.0 (IBM Corporation, Armonk, NY, USA) and AMOS 23.0 (IBM, New York, NY, USA) and G*Power 3.1 (Universität Düsseldorf: Psychologie-HHU). The results were assessed using a significance level of 0.05 [69]. Power analysis was employed to ascertain the magnitude of the sample size [70]. The data underwent normality tests, and pairwise comparisons of normally distributed data were conducted using the Independent T-Test [62, 71]. The Pearson Correlation test and Linear Regression analysis were employed to investigate the correlation and impact between continuous data [72]. The main influences on quality of life students’ and the path relationships between them were explored through structural equation modeling (SEM), and the following goodness-of-fit indices were used to evaluate the model: λ^2^/df < 5, CFI > 0.90, GFI > 0.90, AGFI > 0.90, IFI > 0.90, and RMSEA < 0.05 [62].

## Results

The study utilizin a T-Test for two independent groups at a significance level of α = 0.05 to assess if there was a significant difference in the levels of free time management, leisure satisfaction, and quality of life among university students based on their gender. The results are presented in Table 1. Test results indicate that there were no significant differences in free time management (t_(211)_ = 0.367; *p* = 0.714) and leisure satisfaction (t_(211)_ = 0.193; *p* = 0.847) based on gender. However, a significant difference was observed in the quality of life (t_(211)_ = 4.189; *p* = 0.000). Males scored significantly higher than females in quality of life.

**Table 1:** Students’ free time management, leisure satisfaction and quality of life by gender

Parameter	Gender	N	M	SD	Df	t	p	d
**Free Time Management**	Male	142	3.59	0.62	211	0.367	0.714	–
	Female	71	3.55	0.56				
**Leisure Satisfaction**	Male	142	3.44	0.57	211	0.193	0.847	–
	Female	71	3.42	0.56				
**Quality of Life**	Male	142	48.77	6.28	211	4.189	0.000**	0.609
	Female	71	44.89	6.56				

**p < 0.01, **N Number of participants, *M* Mean, *SD* Standard deviation, *Df* Degree of freedom, *d* Cohen’s D

Upon analysing the effect dimensions, it was found that gender had a moderate impact on quality of life.

Table 2 shows the Pearson correlation test results applied to determine whether there is of a correlation between age and the duration of activity participation, as well as free time management, leisure satisfaction, and quality of life among university students. The test results revealed a significant positive correlation among age and both free time management (*r* = 0.261; *p* = 0.000) and quality of life (*r* = 0.138; *p* = 0.038). Moreover, there was a positive correlation between the number of days of activity participation and both free time management (*r* = 0.294; *p* = 0.000) and quality of life (*r* = 0.189; *p* = 0.006).

**Table 2:** The relationship between students’ age and number of days of activity participation with free time management, leisure satisfaction and quality of life

Parameter	Value	Free Time Management	Leisure Satisfaction	Quality of Life
**Age**	r	.261^**^	0.109	.138^*^
	p	0.000	0.112	0.038
**Number of Days of Participation in the Activity**	r	.294^**^	0.090	.189^**^
	p	0.000	0.193	0.006

*p < 0.05, **p < 0.01

In Table 3, a linear regression model was constructed to predict quality of life as a function of free time management and leisure satisfaction, and leisure satisfaction as a function of free time management. The regression model calculated for free time management and quality of life (F_(1.211)_ = 51.500; *p* = 0.000), leisure satisfaction and quality of life (F_(1.211)_ = 8.899; *p* = 0.003), free time management and leisure satisfaction (F_(1.211)_ = 16.735; *p* = 0.000) was statistically significant. Free time management explains 19% of quality of life (R = 0.443; R^2^ = 0.196) and 7% of leisure satisfaction (R = 0.271; R^2^ = 0.073). Leisure satisfaction explains 4% of quality of life (R = 0.201; R^2^ = 0.040). It was determined that a one unit increase in free time management resulted in a 4.904 unit increase in quality of life, a 0.288 unit increase in leisure satisfaction, and a one unit increase in leisure satisfaction resulted in a 2.367 unit increase in quality of life.

**Table 3:** The relationship between students’ free time management, leisure satisfaction and quality of life

Predictor Variable	Predicted Variable	B	Std. Error	(β)	t	R	R^2^	F	p
Free Time Management	Quality of Life	4.907	0.684	0.443	7.176	0.443	0.196	51.500	0.000**
Leisure Satisfaction	Quality of Life	2.367	0.793	0.201	2.983	0.201	0.040	8.899	0.003**
Free Time Management	Leisure Satisfaction	0.288	0.070	0.271	4.091	0.271	0.073	16.735	0.000**

**Fig. 2:**
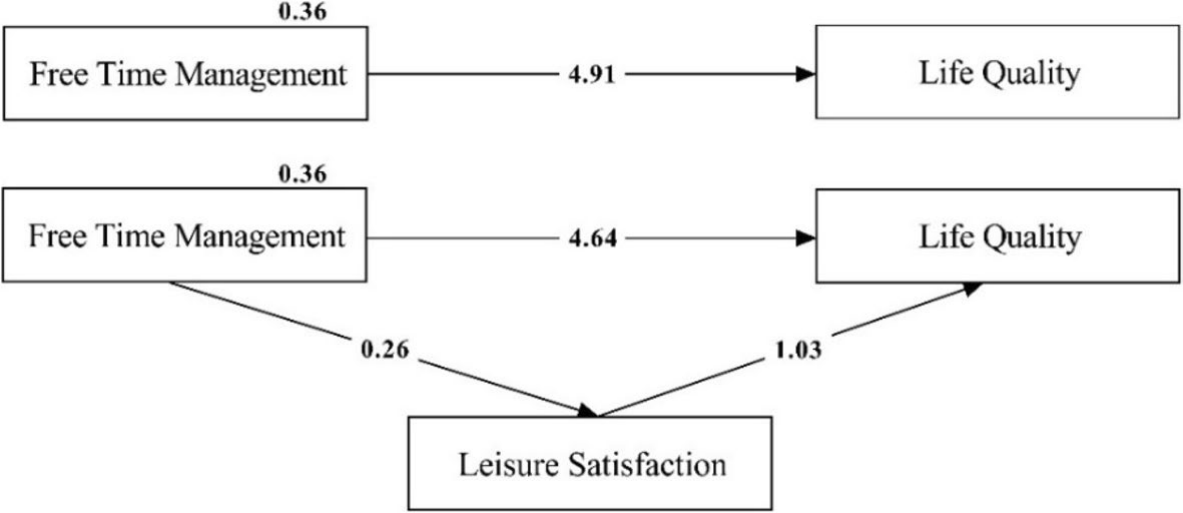
Path diagram for the research model

As shown in Table 4, leisure satisfaction is significantly predicted by free time management (=.26; p.01). Free time management is also a significant predictor of quality of life (= 4.90; p.01). The findings also indicate that leisure satisfaction is a significant predictor of quality of life (= 2.36; p.01). Based on these findings, all the requirements for the mediation effect test are met. Path analysis was used to test the mediating effect of leisure satisfaction in the relationship between free time management and quality of life after the preconditions were met. The results are shown in Fig. 2.

**Table 4:** The mediating effect of leisure satisfaction on the relationship between free time management and quality of life

Structural Pathways		Variables	β	t
**Direct Effects**				
Free Time Management	**→**	Leisure Satisfaction	0.26	4.10**
Free Time Management	**→**	Quality of Life	4.90	7.19**
Leisure Satisfaction	**→**	Quality of Life	2.36	2.99**
**Indirect Effects**				
Free Time Management	**→**	Quality of Life	4.64	6.58**

*p < 0.05

According to the mediating model in Fig. 2, the predictive power of free time management on quality of life decreased from 4.91 to 4.64 in the structural equation modelling in which the mediating effect of free time in the relationship between free time management and quality of life is tested. As a result, it can be stated that leisure satisfaction has a partial mediating effect on the relationship between quality of life and free time management.

## Discussion and implication

In this study, we compared university students’ free time management, leisure satisfaction, and quality of life levels across gender, age, and physical activity participation status. According to the findings, the students’ free time management and leisure satisfaction levels did not differ by gender, whereas their quality of life levels showed a significant difference in favour of males. It is thought that there is no difference between genders in free time management and leisure satisfaction because university students have similar free time levels, whereas the difference in quality of life in favour of males is thought to be due to the expectations and responsibilities defined as gender roles and imposed on women by societies, and this situation puts more pressure on female students. Similarly, in international studies conducted on students, it was observed that female students presented lower quality of life scores in physical and psychological dimensions than male students [28–30, 61, 73]. Furthermore, considering the effect of physical activity on quality of life, it can be said that this situation affects women’s quality of life [27]. However, it should be noted that the findings obtained in terms of gender may differ depending on different regions, age groups, and socioeconomic environments.

The study found a positive correlation between the students’ age, number of activity participation, and their levels of free time management and quality of life. These levels increased in tandem. The effectiveness of age and level of activity participation on free time management is believed to be attributed to the improved utilizing of time through experience. Moreover, individuals who show superior time management skills can allocate more personal time by eliminating the chaotic and frenzied aspects of their daily routine, enabling them to exert control over their level of engagement in desired activities. By engaging in more physical activities and effectively managing time as one ages, individuals can potentially enhance their quality of life, particularly in terms of physical well-being. Demir & Alpullu [59] conducted a study which revealed that age and the extent of activity participation significantly influenced the management of free time. Consistent findings were noted in other investigations [74]. It is thought that the lack of difference in leisure satisfaction between those who participate in physical activity and those who do not participate in physical activity is due to the fact that students are satisfied with the activities they prefer in their free time (even if there is no physical activity), and the difference in favour of those who participate in physical activity in free time management is because physical activity increases physical attractiveness. Similarly, students who participated in physical activity had high scores in free time management and quality of life than students who did not participate in physical activity, according to international studies. According to Mokhtari et al. [75], a sedentary lifestyle has a negative impact on the ability to use time in an international study conducted on students. Other studies on student samples found that physical activity positively influenced free time management [59, 63, 76]. Furthermore, it is reported in foreign sources examining the effect of physical activity on quality of life that participating in physical activity increases one’s level of well-being [77].

The study found a positive correlation between students’ ability to manage their free time, their satisfaction with leisure activities, and their quality of life. Additionally, it was observed that improvements in free time management and leisure satisfaction had a positive impact on students’ quality of life. The effectiveness of free time management on leisure satisfaction and quality of life is believed to stem from the fact that individuals who excel in managing their free time enhance their quality of life through increased engagement in various activities. Moreover, it is believed that individuals who effectively strategize and oversee their leisure activities enhance their enjoyment of free time, thereby positively impacting their quality of life (Fig. 2). Research conducted abroad has demonstrated that effectively managing one’s free time can enhance individuals’ quality of life [56, 78]. Prior research [3, 79] has demonstrated that leisure satisfaction significantly impacts individuals’ quality of life, specifically in relation to their physical health and mental well-being.

Research has demonstrated that engaging in leisure activities typically enhances individuals’ overall well-being and contentment. Lee et al. [80] discovered that engagement in recreational pursuits and the experience of ennui during free time significantly impact one’s overall state of happiness and satisfaction. Trenberth [81] proposed that providing education and counselling to individuals regarding time management and leisure planning can facilitate the development of these skills and enhance their physical and mental well-being. Several studies conducted on elderly individuals have discovered that utilizing their free time for physical activity, social engagement, and leisure pursuits contributes to a sense of group affiliation and social assistance, enhanced mental and physical well-being, and an elevated standard of living [77]. Spiers and Walker [23] discovered that contentment with free time has a substantial impact on happiness, tranquilly, and overall well-being. Regarding the enjoyment of free time, a study by Mannell et al. [31] discovered a positive correlation with overall well-being. In their study, Spiers and Walker [23] discovered that leisure satisfaction had a significant impact on nine aspects of quality of life. These aspects include happiness, peace of mind, living standards, health, achievement, personal relationships, safety, community involvement, future security, and spirituality or religion [82] investigated the associations between leisure satisfaction and quality of life among individuals who participate in badminton, and discovered significant correlations between these two variables. In contrast, Tseng et al. [83] discovered that an individual’s socio-economic status has an impact on their level of satisfaction with leisure activities and quality of life.

When examining the relationship between quality of life and free time management, it was observed that satisfaction with free time partially mediates this relationship. It can be asserted that effectively managing free time is crucial for enhancing the quality of life and ensuring the satisfaction of individuals. Put simply, it has been noted that individuals who effectively manage their free time and engage in activities that bring them satisfaction play a significant role in the connection between free time management and quality of life. This implies that leisure time management serves as a mediating variable in the relationship between leisure satisfaction and quality of life, suggesting that although leisure time management does not have a direct impact on quality of life, it functions as an important intermediary variable influencing the relationship between these two variables. Effective management of leisure time can contribute to individuals feeling more satisfied with themselves. Personal satisfaction can increase when individuals fill their leisure time with activities that are satisfying and meaningful, indirectly enhancing their quality of life. This finding underscores the significance of leisure time management as an influential factor in quality of life, even if it is not directly linked to it. Thus, efficient leisure time management can affect various other factors that contribute to quality of life. These insights can assist individuals in understanding how to manage their leisure time effectively. Engaging in fulfilling and meaningful activities during leisure time can enhance overall life satisfaction and consequently improve quality of life. Moreover, existing literature demonstrates that these two concepts exert a substantial impact on the quality of life, as evidenced by studies conducted by Chick et al. [79], Chizari et al. [78], and Zhou et al. [3].

A study has investigated the impact of leisure time management on quality of life and examined the effects of leisure activities on personal satisfaction [84]. Findings suggest that leisure activities enhance individuals’ levels of personal satisfaction and consequently improve quality of life. However, it is proposed that this effect occurs through the effective management of leisure time [84]. Another research endeavor has explored the influence of leisure activities on quality of life [85]. Results indicate that personal satisfaction increases as a result of participation in leisure activities, positively affecting quality of life, which is closely linked to effective leisure time management [85]. Another study has investigated how effectively managing leisure time affects individuals’ quality of life [86]. Findings demonstrate that consciously utilizing time enhances individuals’ quality of life and consequently elevates their levels of personal satisfaction, highlighting the indirect influence of leisure time management skills on quality of life [86]. A researcher has examined the influence of leisure activities on quality of life [87]. Results show that engagement in active and social leisure activities enhances individuals’ quality of life and increases their levels of personal satisfaction [87]. In a study, the impact of effective leisure time management on individuals’ quality of life was investigated [37]. Results indicate that effective leisure time management enhances individuals’ quality of life and improves their levels of personal satisfaction [37]. Finally, a study has explored the effect of effectively utilizing leisure time on quality of life [88]. Findings reveal that effective leisure time management enhances individuals’ quality of life and increases overall life satisfaction [88].

## Conclusion

In contrast to the existing literature, our study revealed that the interplay between free time management, leisure satisfaction, and quality of life is influenced by gender, age, and participation in physical activity. Furthermore, it was established that the level of contentment with one’s free time served as a mediator in the connection between the overall well-being and the management of free time. Consequently, it was noted that males exhibited a superior standard of living, and the accumulation of life experience and engagement in physical activity positively influenced both the ability to manage free time and quality of life. It has been observed that students who effectively organize and manage their leisure experience an improvement in their overall well-being through an increased enjoyment derived from free time activities, as opposed to those who lack proficiency in managing their free time. The satisfaction of individuals who effectively managed their free time was found to be a crucial factor in this relationship. In this scenario, students who are unable to effectively regulate their free time may transform into individuals who struggle to prioritize their tasks, meet deadlines, experience elevated stress levels, and achieve low levels of success. Individuals who encounter physical, physiological, and psychological issues may diminish their contentment with both free time and overall life. Providing training and counselling to university students, particularly those who are ambitious about their future, on free time management and planning can potentially enhance their quality of life and satisfaction with leisure activities. For this purpose:

- Universities can arrange for experts to visit and enhance students’ understanding of time management skills, encouraging them to apply these skills in their daily lives.
- This study specifically focuses on university students and does not include other groups. These concepts can be collectively analyzed in various demographic groups, including the elderly, individuals with disabilities, and immigrants.

### Limitations

Some limitations are included in the results of this study: To begin, the research used a quantitative approach. The research was conducted using a simple random sampling method. This indicates that generalizations may be limited, and the sample may not fully represent the population. The data used in the study were largely provided by the participants themselves, which could introduce self-reporting biases and inaccuracies. The scales used in the study are adapted versions of scales available in the literature. However, it should be noted that these adaptations may be influenced by language and cultural differences, requiring additional attention to ensure the full accuracy of measurements. The research is a cross-sectional study conducted at a specific point in time. This design may limit the ability to determine variability over time or causality in the correlation. For future research, longitudinal or experimental designs could provide more robust results. SEM demands meticulous variable selection and the acknowledgment of measurement errors. Erroneous variable choices or measurement inaccuracies could compromise the model’s fidelity. SEM offers a means to scrutinize intricate relationships. Nonetheless, the formulation and interpretation of complex models pose challenges. Although the complexity of the model employed in this study is modest, it may constitute a constraint for researchers aiming to delve into more intricate relationships. The study focused on specific demographic characteristics (gender, age, participation in physical activity). However, the neglect of other potential factors may hinder a comprehensive analysis of the results. Considering these limitations provides a more balanced perspective on interpreting the results and their generalizability. Future research should address these limitations to further advance our understanding of the topic.

## Data Availability

No datasets were generated or analysed during the current study.
